# Abdominal symptoms as initial manifestation of COVID-19: a case series

**DOI:** 10.31744/einstein_journal/2020RC5831

**Published:** 2020-10-09

**Authors:** Lucas Tadashi Wada Amaral, Vanessa Mizubuti Brito, Gabriel Laverdi Beraldo, Eduardo Kaiser Ururahy Nunes Fonseca, Patrícia Yokoo, Aley Talans, Marcelo Oranges, Rodrigo Caruso Chate, Ronaldo Hueb Baroni, Gilberto Szarf

**Affiliations:** 1 Hospital Israelita Albert Einstein São PauloSP Brazil Hospital Israelita Albert Einstein , São Paulo , SP , Brazil .

**Keywords:** COVID-19, Coronavirus infections, Computed tomography, X-ray computed, Thorax/diagnostic imaging, Gastrointestinal diseases/diagnostic imaging, Abdomen/diagnostic imaging

## Abstract

The COVID-19 became a pandemic in early 2020. It was found, at first, that the main manifestations of this new virus occur through respiratory and constitutional symptoms. Therefore, chest tomography was elected as the best imaging test to assess the extent of pulmonary involvement and as a good prognostic predictor for the disease. However, as new studies were produced, the gastrointestinal involvement of COVID-19 becomes more evident, with reports from patients who manifested mainly or only gastrointestinal symptoms in the course of the disease. Thus, in some cases, the initial investigation is carried out at the emergency department with an abdominal computed tomography. We report a case series of ten patients who came to the emergency department of our institution with a chief gastrointestinal complaint, and were initially submitted to an abdominal computed tomography as the first investigation. Although most of the patients did not have significant changes in the abdominal images, most reported patients had pulmonary findings visualized at the lung bases, which were later designated as typical COVID-19 pulmonary findings on chest computed tomography. Only one patient had atypical COVID-19 lung changes on chest computed tomography. All patients had a positive real-time polymerase chain reaction for COVID-19. It is imperative to alert radiologists, especially abdominal radiologists, with the possibility of COVID-19 isolated gastrointestinal symptoms. Besides, it must become a habit to radiologists to assess the pulmonary basis on abdominal scans, a site commonly affected by the new coronavirus.

## INTRODUCTION

The new coronavirus disease (COVID-19) was initially described in December 2019 in Wuhan (Hubei, China), rapidly spread worldwide and was classified as pandemic by the World Health Organization (WHO), on March 11, 2020. ^( 1 )^

So far, the primordial measures against this new agent are early detection and isolation of suspected patients. The most common initial symptoms described for the COVID-19 infection include constitutional and respiratory symptoms, such as fever, malaise, cough, coryza, and dyspnea. ^( 2 )^

Recent studies showed that the new coronavirus, an RNA virus, uses the angiotensin-converting enzyme 2 (ACE2) to enter the cells, yielding potential to infect different organs and systems of the human body. ^( 3 , 4 )^ This mechanism may explain the occurrence of gastrointestinal symptoms in patients with COVID-19, such as diarrhea, nausea, vomits, and lack of appetite, who may or may not be present with respiratory symptoms. However, it was observed that some patients are asymptomatic from the respiratory point of view, and have only abdominal complaints as their initial clinical findings. This phenomenon can be a diagnostic challenge and a potential risk of COVID-19 transmission, not only to other patients but also to the health professionals involved in healthcare.

Therefore, it is important for the abdominal radiologists, radiologists on-call, and other physicians that are on the frontline against the COVID-19, to be aware of the importance of evaluating the lung bases on abdominal computed tomography (CT) in this present pandemic, even in the absence of respiratory complaints.

## CLINICAL PRESENTATION

We retrospectively analyzed all emergency abdominal CT of our institution performed between March 15, 2020 and April 21, 2020, looking for changes caused by COVID-19 on the pulmonary basis included on abdominal images, which could lead to further investigation for this viral pneumonia.

Ten patients met these inclusion criteria, and we further reviewed their past medical history.

Of the patients assessed, five were male (50%). The mean age was 62 years, ranging from 41 to 84 years. All ten patients tested positive for COVID-19 in real-time polymerase chain reaction (RT-PCR), obtained from an nasopharyngeal swab sample.

The most frequent gastrointestinal symptoms were abdominal pain, diarrhea, nausea, vomiting, and lack of appetite ( Table 1 ), in agreement with other studies in the literature. ^( 5 , 6 )^ All patients analyzed had gastrointestinal symptoms that preceded the respiratory symptoms.

**Table 1:** Gastrointestinal symptoms

Pacient	Sex	Age	Abdominal pain	Diarrhea	Nausea/vomiting	Lack of appetite
1	M	84	+	+	-	+
2	F	52	+	-	-	-
3	M	72	+	-	-	+
4	F	73	+	+	-	-
5	F	75	-	+	-	-
6	M	76	+	+	+	+
7	M	41	+	+	-	-
8	M	77	+	+	+	+
9	F	56	-	+	+	-
10	F	22	+	+	+	+

M: male; F: female.

Abdominal pain was the most prevalent complaint in the patients assessed; - two presented with diffuse abdominal pain and four with epigastric pain. One patient had pain in the left flank, and another had pain in the right iliac fossa.

Eight patients presented with diarrhea, with a mean duration of 7 days, range of 3 to 20 days. The patient with history of diarrhea for 20 days stayed longer at the hospital, took several antibiotics, which may have contributed to longer duration of this symptom.

**Figure 1:**
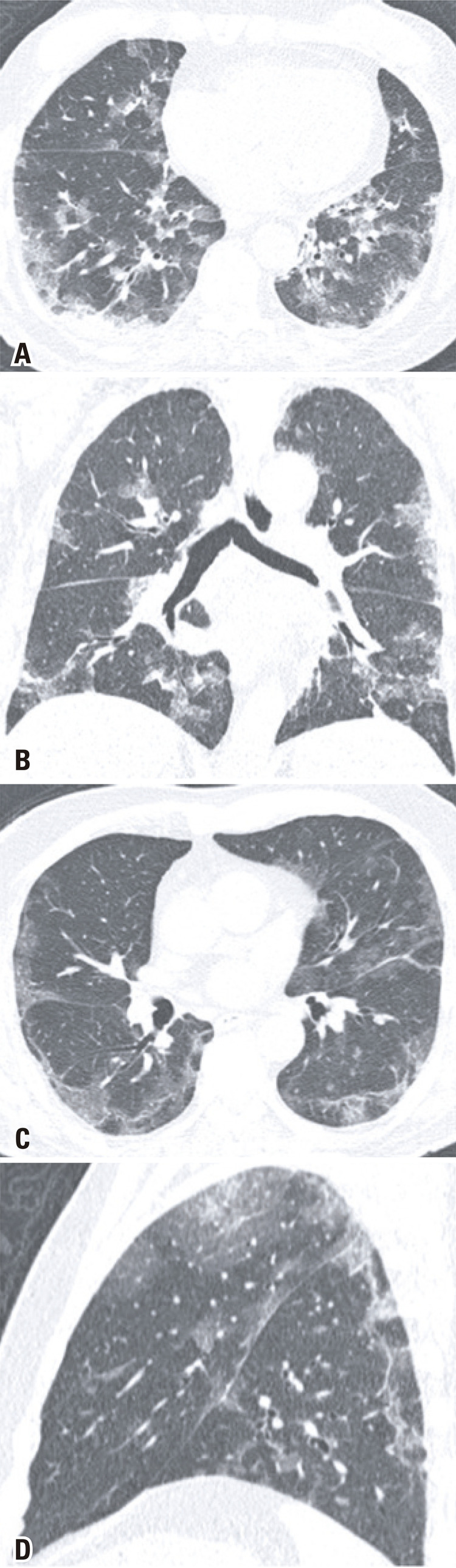
Axial (A, C), coronal (B) and sagital (D) images of chest computed tomography showing typical COVID-19 pulmonary findings

**Figure 2:**
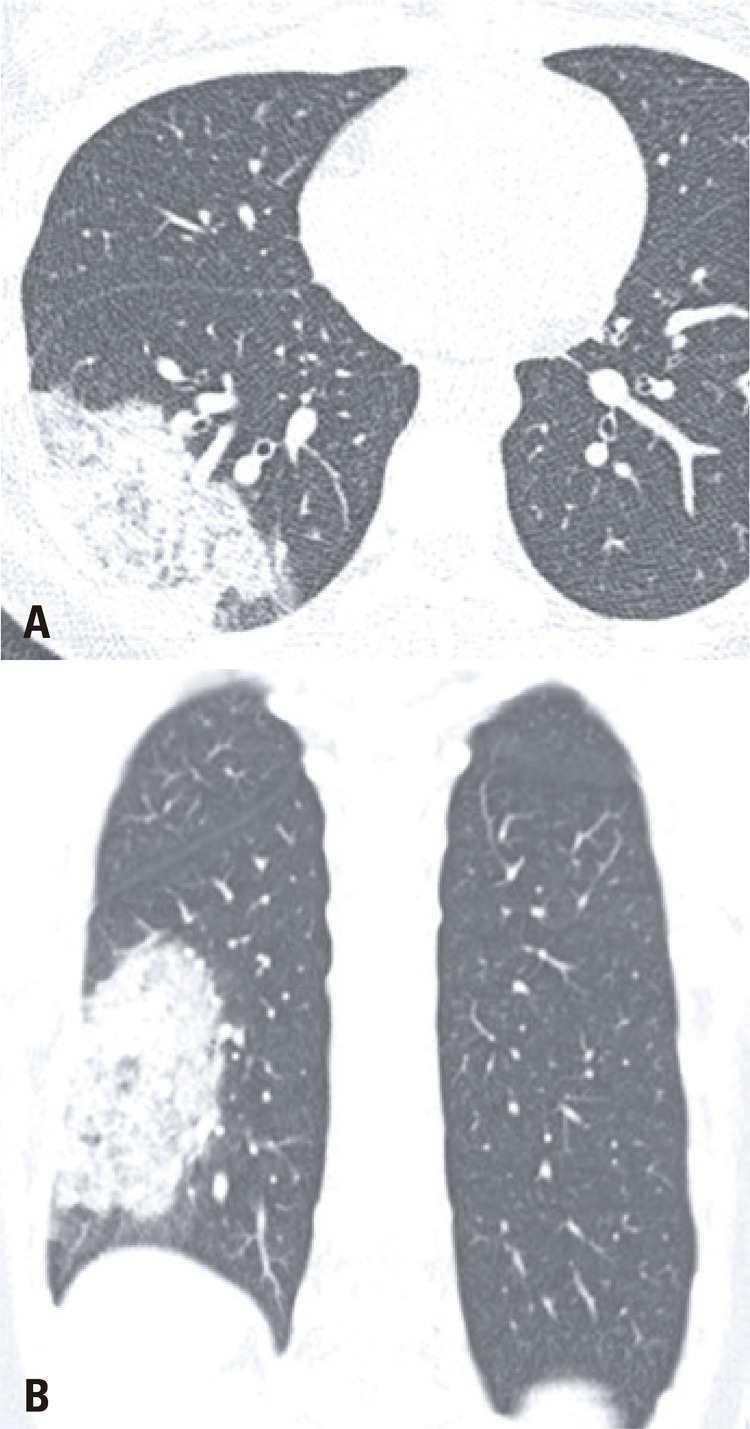
Axial (A) and coronal (B) chest computed tomography images show an unique pulmonary consolidation in the right lower lobe, an atypical finding in COVID-19

Since the chief complaint of the analyzed patients was related to gastrointestinal symptoms, the investigation initiated with an abdominal CT exam, and 80% (8/10) of the exams had no significant abdominal changes. Two CT had positive findings. Nine out of ten of the subsequent chest CT, all of which were motivated by the initial abdominal CT findings had typical COVID-19 alterations, ^( 7 )^ such as peripheral and basal predominant ground-glass opacities, with septal thickening and thin reticulation, sparse consolidations and subpleural curvilinear lines ( Figure 1 ). One chest CT demonstrated atypical COVID-19 findings, characterized by a unique alveolar consolidation in the right lower lobe ( Figure 2 ).

## CASE REPORTS

### First case

**Figure 3:**
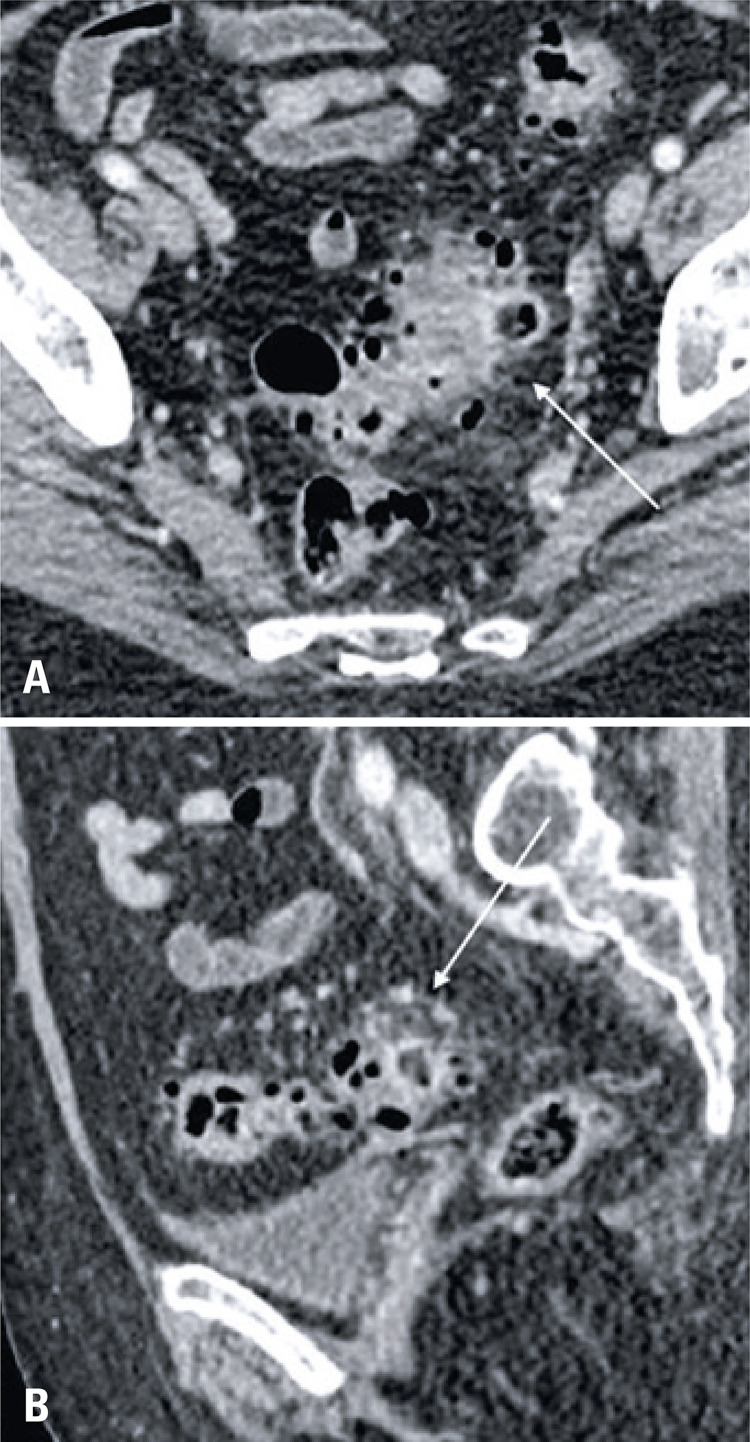
Axial (A) and sagital (B) images of an abdomen computed tomography illustrates multiple diverticula in the sigmoid colon. One showed thickened walls (arrows) with adjacent fat stranding, findings consistent with acute diverticulitis

A 74-year-old female patient, presented to the emergency department on March 18, 2020, with a history of abdominal pain, on the right iliac fossa, for 15 days. She referred fever for 4 days, and denied having diarrhea, nausea, vomiting, or respiratory symptoms. She presented diffuse abdominal pain upon palpation, more intense on the right iliac fossa. Her chest auscultation was unremarkable. The patient was submitted to a contrast-enhanced CT of the abdomen, with findings consistent with non-complicated acute diverticulitis in the sigmoid colon ( Figure 3 ). She received analgesics and antibiotics and was discharged.

**Figure 4:**
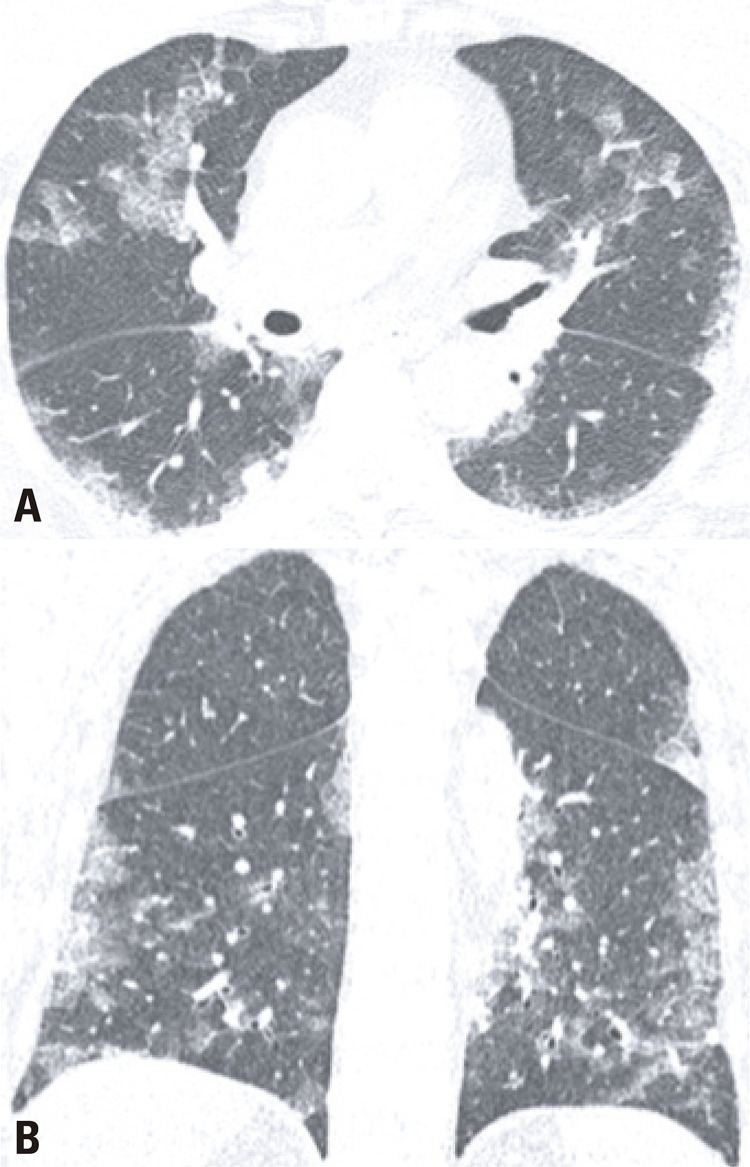
Axial (A) and coronal (B) images of a chest computed tomography illustrate multiple and bilateral ground glass opacities, septal thickening and reticulation, findings consistent with COVID-19

**Figure 5:**
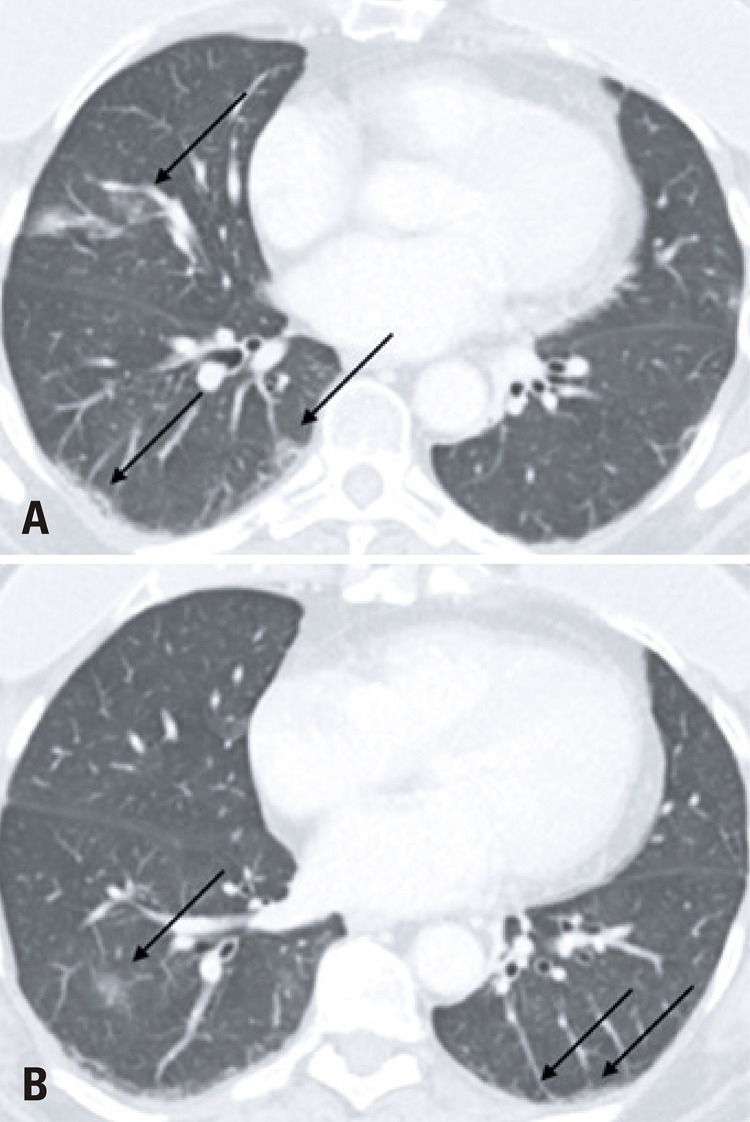
Axial images (A and B) of the lung basis in an abdomen computed tomography show subtle ground glass opacities with some septal thickening, which possibly represented incipient COVID-19 changes (arrows)

After 4 days of antibiotics, the patient returned to the emergency department complaining of weakness, abdominal cramps, and lack of appetite, still with no respiratory symptoms. She was admitted to the hospital, and presented diarrhea, cough, and desaturation. A RT-PCR was requested from the oropharyngeal swab, and it was positive for COVID-19. A chest CT was performed on March 23, 2020, and showed typical findings for COVID-19 ( Figure 4 ). A retrospective analysis of the lung basis on the abdomen CT on March 18, 2020 revealed discrete ground-glass opacities with areas of thin reticulation and septal thickening on the periphery of the medium lobe and on the posterior basal segments of both lungs, findings that possibly were related to incipient COVID-19 changes ( Figure 5 ). The patient had a good progression, being discharged home on March 31, 2020, in a good general status.

### Second case

**Figure 6:**
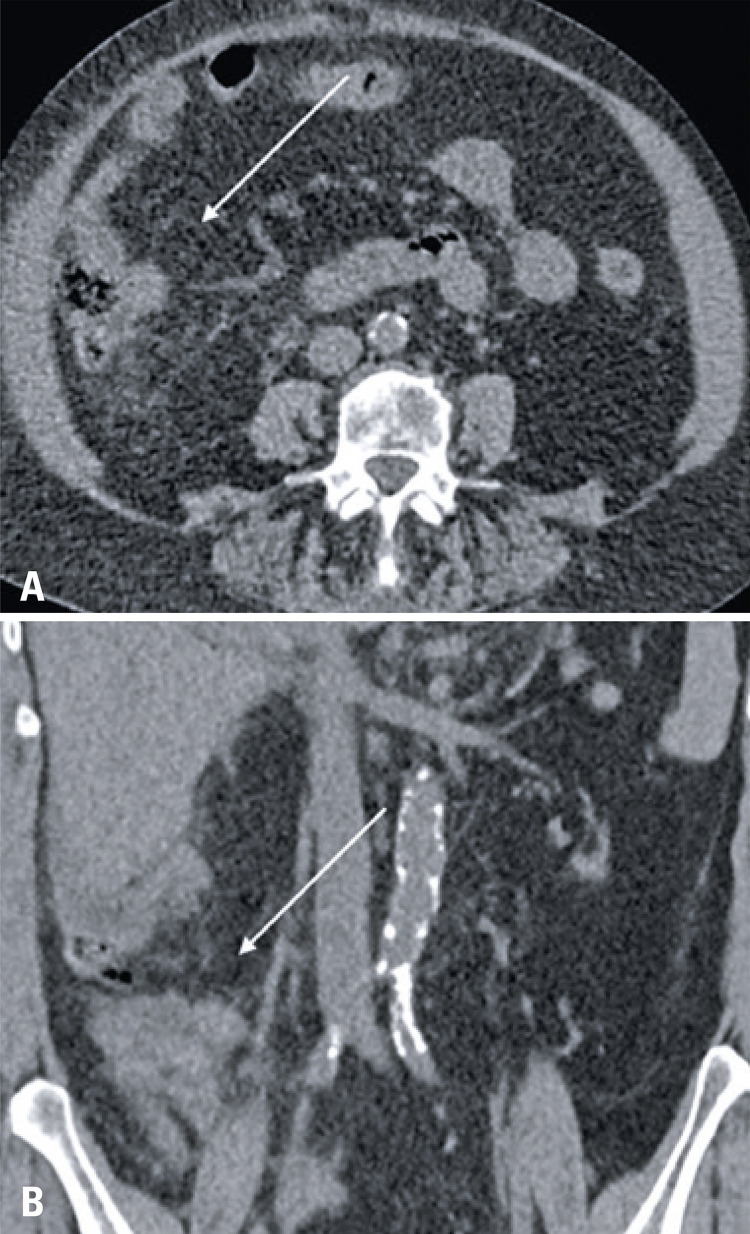
Axial (A) and coronal (B) images of a non-enhanced abdomen computed tomography show thickened walls in the cecum and terminal ileum, with fat stranding (arrows), consistent with enterocolitis

**Figure 7:**
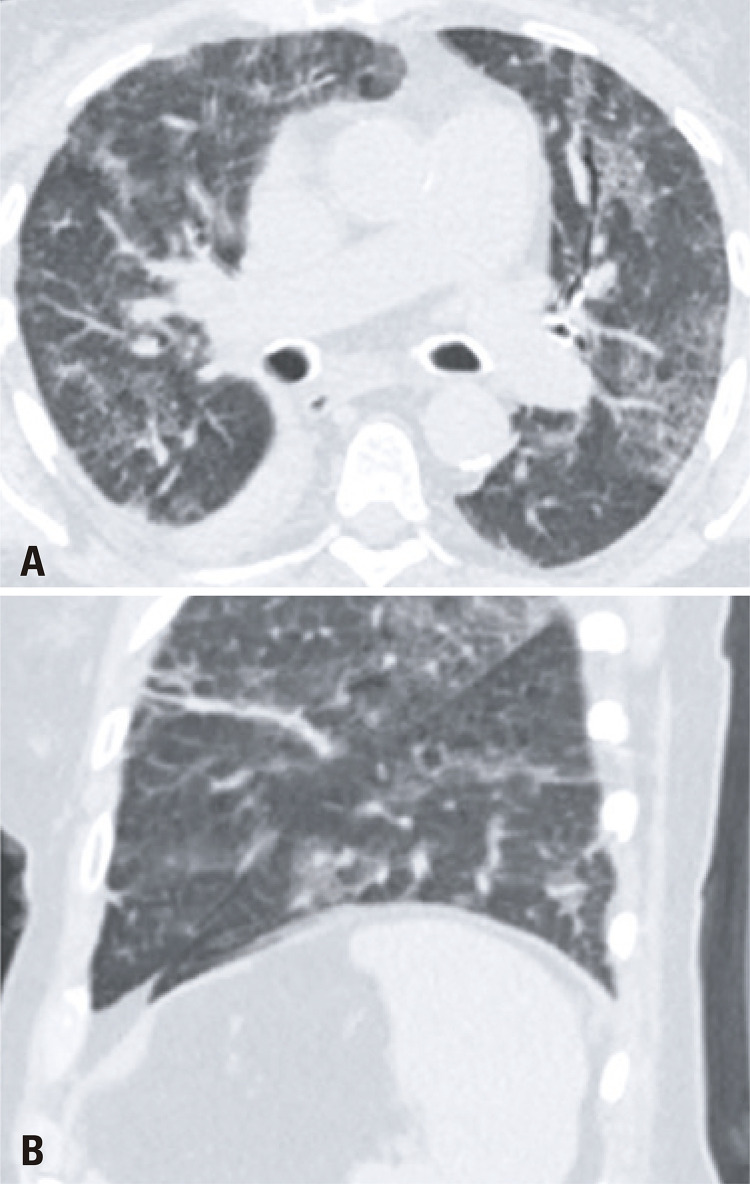
Axial (A) and sagital (B) images of the lung basis in an abdomen computed tomography show ground-glass opacities with septal thickening and fine reticulation, typical COVID-19 pulmonary findings

A 75-year-old female patient came to the emergency department on March 18, 2020, presenting with malaise, fever, diarrhea, and dyspnea. The hypothesis of an abdominal sepsis was raised, and the patient was admitted to an intensive care unit. An abdominal CT with no contrast enhancement was requested, which revealed a thickened ascending colon and distal ileum, associated with adjacent fat stranding, findings that suggested enterocolitis ( Figure 6 ). In the same exam, on the pulmonary basis, areas of peripheral ground-glass opacities were observed on both lungs, especially on the left, and pleural effusion on the right lung ( Figure 7 ). These changes led to the request of a chest CT, which had findings consistent with viral pneumonia (typical of COVID-19). With this suspect, RT-PCR was done and it returned positive. The patient progressed with severe respiratory failure and was intubated. She had a slow but steady recovery, being discharged from the hospital after one month.

## DISCUSSION

As the COVID-19 spreads and new studies are finalized, its gastrointestinal effects become more evident: some symptoms, such as abdominal pain, diarrhea, nausea, and vomiting are not rare, as deemed in the beginning of the pandemic. A study by Lin et al., ^( 6 )^ reported a 61% prevalence of gastrointestinal symptoms upon admission or in the course of the disease.

We illustrated one case of acute diverticulitis that preceded a new coronavirus infection, and another presenting with diarrhea and signs of enterocolitis during de course of the disease. We questioned if the findings of enterocolitis could be a manifestation of COVID-19 infecting enterocytes, or if there was an intestinal coinfection.

New evidence in the literature suggests that there is ACE2 expression in the enterocytes, ^( 4 , 8 )^ acting as an inflammatory mediator. Besides, new studies found the virus in feces of infected patients, supporting not only the possibility of direct intestinal infection but also the possibility of a fecal-oral transmission route. ^( 8 , 9 )^

Abdominal complaints are frequently assessed with imaging studies, and some protocols include images of the pulmonary bases, which are frequent sites of involvement by COVID-19. We believe that some COVID-19 patients will not show respiratory symptoms, leading to a challenging diagnosis, delaying adequate isolation measurements. In addition, some studies have demonstrated that abdominal symptoms are not rare in this group of patients and can appear earlier in the course of the disease. ^( 10 , 11 )^ Therefore, in the actual pandemic, it is of paramount importance that radiologists keep a high grade of suspicion even when analyzing an exam not directed to the chest, and even when there is no suspicion by the clinical staff, assuring a prompt COVID-19 diagnosis. Since there is no specific treatment for COVID-19, the early diagnosis has an impact on the medical care concerning isolation, reducing transmissibility of the disease not only at home but also at hospitals.

## CONCLUSION

COVID-19 has a broad spectrum of gastrointestinal symptoms, which are much common than we originally considered. In this pandemic context, we believe radiologists, especially abdominal radiologists, should be aware of the typical and atypical pulmonary changes of coronavirus disease when assessing the lung bases.
