# Supplementary material for: Abdominal symptoms as initial manifestation of COVID-19: a case series
Source: Einstein (Sao Paulo). 2020 Oct 9;18:eRC5831. doi: 10.31744/einstein_journal/2020RC5831 (PMC7546681; doi:10.31744/einstein_journal/2020RC5831)
Supplement: Translation

# Translation: Sintomas abdominais como manifestação inicial da COVID-19: uma série de casos

**Authors:** Lucas Tadashi Wada Amaral, Vanessa Mizubuti Brito, Gabriel Laverdi Beraldo, Eduardo Kaiser Ururahy Nunes Fonseca, Patrícia Yokoo, Aley Talans, Marcelo Oranges, Rodrigo Caruso Chate, Ronaldo Hueb Baroni, Gilberto Szarf

**Affiliations:** 1 Brasil Hospital Israelita Albert Einstein, São Paulo, SP, Brasil .

**Keywords:** COVID-19, Infecções por coronavírus, Tomografia computadorizada por raios X, Tórax/diagnóstico por imagem, Gastroenteropatias/diagnóstico por imagem, Abdome/diagnóstico por imagem

## Abstract

A COVID-19 foi declarada uma pandemia no início de 2020. Constatou-se, inicialmente, que as principais manifestações desse novo vírus ocorrem por meio de sintomas respiratórios e constitucionais. A tomografia do tórax foi eleita o exame de imagem para avaliar a extensão do comprometimento pulmonar e como um fator preditivo do prognóstico para a doença. No entanto, à medida que novos estudos são produzidos, o envolvimento gastrointestinal da COVID-19 torna-se mais evidente, com relatos de pacientes que manifestaram principalmente ou apenas sintomas gastrointestinais no decorrer da doença. Em alguns casos, a investigação inicial é realizada no pronto-socorro, com tomografia computadorizada do abdome. Relatamos uma série de casos de dez pacientes que compareceram ao serviço de emergência da instituição com uma queixa principal gastrointestinal e foram submetidos inicialmente a uma tomografia computadorizada de abdome como primeira investigação. Embora a maioria dos pacientes não tenha apresentado alterações significativas nas imagens abdominais, eles apresentaram achados pulmonares visualizados nas bases pulmonares, que depois foram caracterizadas como achados pulmonares típicos de COVID-19 nas tomografias de tórax subsequentes. Apenas um paciente apresentou achados atípicos para COVID-19 na tomografia. Todos os pacientes tiveram reação em cadeia da polimerase em tempo real positiva para o novo coronavírus. É muito importante alertar os radiologistas, principalmente os radiologistas abdominais, da possibilidade de sintomas gastrointestinais isolados no contexto da COVID-19. Além disso, deve ser um hábito para todos os radiologistas avaliar as bases pulmonares nas tomografias de abdome, local comumente afetado pela COVID-19.

## INTRODUÇÃO

A doença do novo coronavírus (COVID-19) foi inicialmente descrita em dezembro de 2019 em Wuhan, Hubei, na China. Ela se disseminou rapidamente pelo mundo e foi classificada como pandemia pela Organização Mundial de Saúde (OMS) em 11 de março de 2020. ^( 1 )^

Até o momento, as medidas primordiais contra esse novo agente são detecção precoce e isolamento de casos suspeitos. Os sintomas iniciais mais comuns descritos para a COVID-19 incluem sintomas constitucionais e respiratórios, como febre, mal-estar, tosse, coriza e dispneia. ^( 2 )^

Estudos recentes mostraram que o COVID-19, um vírus de RNA, utiliza a enzima conversora de angiotensina 2 (ECA2) para entrar nas células, o que lhe confere o potencial de infectar diferentes órgãos e sistemas do corpo humano. ^( 3 , 4 )^ Esse mecanismo pode explicar a ocorrência de sintomas gastrointestinais em pacientes com infecção pela COVID-19, como diarreia, náusea, vômitos e inapetência, que podem ou não se manifestar juntamente de sintomas respiratórios. Porém, observou-se que alguns pacientes são assintomáticos do ponto de vista respiratório e apresentam apenas queixas abdominais como achado clínico inicial. Esse fenômeno pode representar um desafio diagnóstico e um risco potencial de transmissão da COVID-19, não apenas a outros pacientes, mas também aos profissionais de saúde envolvidos no atendimento.

Assim, é importante alertar os radiologistas abdominais, radiologistas de plantão e outros médicos que atuam na linha de frente contra a COVID-19 sobre a importância de se avaliar a base dos pulmões em tomografia computadorizada (TC) de abdome durante a presente pandemia, mesmo na ausência de queixas respiratórias.

## APRESENTAÇÃO CLÍNICA

Analisamos retrospectivamente todas as TC abdominais de emergência da nossa instituição, realizadas entre 15 de março de 2020 e 21 de abril de 2020, em busca de alterações causadas pela COVID-19 nas bases pulmonares contempladas em imagens abdominais, que poderiam motivar uma investigação mais aprofundada para esse tipo de pneumonia viral.

Dez pacientes atenderam aos critérios de inclusão, e analisamos mais a fundo a história pregressa deles.

Dos pacientes avaliados, cinco eram do sexo masculino (50%). A média de idade foi de 62 anos, variando de 41 a 84 anos. Todos os dez pacientes tiveram resultado positivo no teste de reação em cadeia da polimerase em tempo real (RT-PCR) usando amostra de *swab* nasofaríngeo.

Os sintomas gastrointestinais mais frequentes foram dor abdominal, diarreia, náusea, vômitos e inapetência ( Tabela 1 ), em concordância com outros estudos da literatura. ^( 5 , 6 )^ Todos os pacientes analisados apresentaram sintomas gastrointestinais antes dos respiratórios.

**Tabela 1:** Sintomas gastrointestinais

Paciente	Sexo	Idade	Dor abdominal	Diarreia	Náusea/vômitos	Inapetência
1	M	84	+	+	-	+
2	F	52	+	-	-	-
3	M	72	+	-	-	+
4	F	73	+	+	-	-
5	F	75	-	+	-	-
6	M	76	+	+	+	+
7	M	41	+	+	-	-
8	M	77	+	+	+	+
9	F	56	-	+	+	-
10	F	22	+	+	+	+

M: masculino; F: feminino.

Dor abdominal foi a queixa mais prevalente nos pacientes avaliados – dois apresentaram dor abdominal difusa e quatro, epigástrica. Um paciente tinha dor no flanco esquerdo e outro apresentou dor na fossa ilíaca direita.

Oito pacientes apresentaram diarreia, com duração média de 7 dias, variando de 3 a 20 dias. O paciente com história de 20 dias de diarreia permaneceu no hospital por um período mais longo e precisou tomar diversos antibióticos, o que pode ter contribuído para a longa duração desse sintoma.

Como a queixa principal dos pacientes analisados tinha relação com sintomas gastrointestinais, a investigação começou com exame de TC do abdome, e 80% (8/10) dos exames não mostraram alterações abdominais significativas. Duas TC apresentaram achados positivos. Nove das dez TC de tórax subsequentes, todas elas motivadas pelos achados das TC de abdome inicial, revelaram alterações típicas da COVID-19, ^( 7 )^ como opacidades em vidro fosco predominantemente periféricas e basais associadas a espessamento septal e reticulação fina, além de consolidações esparsas e linhas curvilíneas subpleurais ( Figura 1 ). Uma TC de tórax demonstrou achados atípicos de COVID-19, caracterizados por consolidação alveolar única no lobo inferior direito ( Figura 2 ).

**Figura 1:** Imagens axial (A, C), coronal (B) e sagital (D) de tomografia computadorizada de tórax, retratando achados pulmonares comuns da COVID-19

**Figura 2:** Imagens axial (A) e coronal (B) de tomografia computadorizada de tórax ilustram consolidação alveolar no lobo inferior direito, um achado incomum para COVID-19

## RELATOS DE CASOS

### Primeiro caso

Paciente do sexo feminino, 74 anos de idade, compareceu ao pronto-socorro em 18 de março de 2020, com história de dor abdominal na fossa ilíaca direita há 15 dias. Relatou febre por 4 dias e negou outros sintomas. Ao exame físico, apresentou dor abdominal difusa à palpação, mais intensa na fossa ilíaca direita. A ausculta torácica foi normal. A paciente foi submetida à TC abdominal com contraste, com achados condizentes com diverticulite aguda não complicada do cólon sigmoide ( Figura 3 ). Ela foi tratada com analgésicos e antibióticos e recebeu alta.

**Figura 3:** Imagens axial (A) e sagital (B) de tomografia computadorizada do abdome demonstra múltiplos divertículos no cólon sigmoide. Um deles está com paredes espessadas (setas) e estriações da gordura adjacente, achados consistentes com diverticulite aguda

Após 4 dias de antibióticos, a paciente retornou ao pronto-socorro com queixa de fraqueza, cólicas abdominais e inapetência, ainda sem sintomas respiratórios. Foi hospitalizada, após apresentar diarreia, tosse e dessaturação. Foi solicitado o RT-PCR do *swab* orofaríngeo, com resultado positivo para COVID-19. Foi realizada TC do tórax em 23 de março de 2020, que revelou achados típicos de COVID-19 ( Figura 4 ). A análise retrospectiva da base do pulmão da TC de abdome realizada em 18 de março de 2020 revelou tênues opacidades em vidro fosco, com áreas de fina reticulação e espessamento septal na periferia do lobo médio e nos segmentos basais posteriores de ambos os pulmões, achados estes possivelmente relacionados a uma infecção incipiente pelo novo coronavírus ( Figura 5 ). A paciente teve boa evolução e recebeu alta em 31 de março de 2020, em bom estado geral de saúde.

**Figura 4:** Imagens axial (A) e coronal (B) de tomografia computadorizada de tórax ilustram múltiplas opacidades em vidro fosco bilaterais, espessamento septal e fina reticulação, achados típicos de COVID-19

**Figura 5:** Imagens axiais (A e B) das bases pulmonares da primeira tomografia computadorizada do abdome mostram sutis opacidades em vidro fosco arredondadas, com algum espessamento septal, sugestivas de infecção incipiente pela COVID-19 (setas)

### Segundo caso

Paciente do sexo feminino, 75 anos de idade, compareceu ao pronto-socorro em 18 de março de 2020, com mal-estar, febre, diarreia e dispneia. Foi levantada a hipótese de sepse abdominal, e a paciente foi internada em unidade de terapia intensiva. Foi solicitada TC abdominal sem contraste, que revelou espessamento do cólon ascendente e do íleo distal, associado a estriações da gordura adjacente, achados sugestivos de enterocolite ( Figura 6 ). No mesmo exame, observaram-se, nas bases pulmonares, áreas de opacidades em vidro fosco periféricas, principalmente no pulmão esquerdo, e derrame pleural à direita ( Figura 7 ). Essas alterações motivaram a solicitação de uma TC de tórax, que demonstrou achados compatíveis com pneumonia viral (típica da COVID-19). Com essa suspeita, foi realizado o RT-PCR, que retornou positivo. A paciente evoluiu com insuficiência respiratória grave e foi entubada. Ela apresentou lenta, porém contínua recuperação, recebendo alta hospitalar após um mês.

**Figura 6:** Imagens axial (A) e coronal (B) da tomografia computadorizada do abdome sem contraste, mostrando paredes espessadas no ceco e íleo terminal com estriações de gordura (setas), condizentes com enterocolite

**Figura 7:** Imagens axial (A) e sagital (B) das bases pulmonares da tomografia computadorizada de abdome mostram opacidades em vidro fosco com espessamento septal e reticulação fina, achados pulmonares típicos da COVID-19

## DISCUSSÃO

À medida que a COVID-19 se propaga, e novos estudos são finalizados, os efeitos gastrointestinais da doença tornam-se mais evidentes: alguns sintomas, como dor abdominal, diarreia, náusea e vômitos, não são tão incomuns quanto se pensava no início da pandemia. Um estudo de Lin et al., ^( 6 )^ relatou prevalência de 61% de sintomas gastrointestinais na admissão ou no curso da doença.

Ilustramos um caso de diverticulite aguda precedendo infecção por COVID-19, e outro que apresentou diarreia e sinais de enterocolite durante o curso da doença. Questionamos se os achados de enterocolite poderiam ser uma manifestação de infecção dos enterócitos pela COVID-19, ou se haveria uma coinfecção intestinal.

Novas evidências na literatura sugerem que há expressão de ECA2 nos enterócitos ^( 4 , 8 )^ atuando como mediadora inflamatória. Além disso, novos estudos encontraram o vírus nas fezes de pacientes infectados, o que corrobora não apenas a possibilidade de infecção intestinal direta, mas também uma possível via de transmissão fecal-oral. ^( 8 , 9 )^

Queixas abdominais são frequentemente avaliadas com estudos de imagem, e alguns protocolos incluem imagens das bases do pulmão, um local frequentemente acometido pela COVID-19. Acreditamos que alguns pacientes com COVID-19 não apresentam sintomas respiratórios, o que dificulta o diagnóstico, atrasando a implementação de medidas de isolamento adequadas. Além disso, estudos demonstraram que sintomas abdominais não são incomuns nesse grupo de pacientes e podem aparecer precocemente no curso da doença. ^( 10 , 11 )^ Assim, na presente pandemia, é primordial que os radiologistas mantenham um alto nível de suspeição, mesmo para exames que não sejam dirigidos ao tórax e quando não houver uma suspeita clara da equipe clínica, para garantir um diagnóstico rápido da COVID-19. Como ainda não há tratamento específico para a COVID-19, o diagnóstico precoce tem impacto no atendimento médico, particularmente em relação ao isolamento, reduzindo a transmissibilidade da doença não apenas no ambiente domiciliar, mas também nos hospitais.

## CONCLUSÃO

A COVID-19 apresenta um amplo espectro de sintomas gastrointestinais, que são muito mais comuns do que se pensava originalmente. No contexto da presente pandemia, acreditamos que os radiologistas, principalmente os radiologistas abdominais, devem ficar alertas a alterações pulmonares típicas e atípicas da doença do novo coronavírus ao avaliarem a base dos pulmões.
